# Study of Intact Glycosidic Aroma Precursors in Recovered Minority White Grape Varieties under Water Stress Conditions

**DOI:** 10.1021/acs.jafc.5c17579

**Published:** 2026-03-02

**Authors:** Cristina Cebrián-Tarancón, Mirko De Rosso, Annarita Panighel, Riccardo Flamini, Gonzalo L. Alonso, M. Rosario Salinas, A. Sergio Serrano, Rosario Sánchez-Gómez

**Affiliations:** † Cátedra de Química Agrícola, Universidad de Castilla-La Mancha, E.T.S.I. Agronómica y de Montes y Biotecnología (ETSIAMB), Avda. de España s/n, 02071 Albacete, Spain; ‡ Council for Agricultural Research and Economics—Research Center for Viticulture & Enology, Viale XXVIII Aprile 26, Conegliano, Treviso 31015, Italy; § Instituto Regional de Investigación y Desarrollo Agroalimentario y Forestal de Castilla-La Mancha (IRIAF), Ctra. Toledo-Albacete s/n, 13700 Tomelloso, Spain

**Keywords:** glycosidic volatile, Jarrosuelto, Pintada, grape, variety marker, QTOF

## Abstract

The identification of viticultural strategies capable of mitigating the effects of climate change, including increased temperatures and reduced water availability, has prompted renewed interest in minor grapevine varieties adapted to specific growing areas. The enological suitability of these cultivars depends, in part, on the characterization of their glycosylated aroma precursors. In this study (2022 harvest), two recovered white grape varieties from Castilla–La Mancha, Pintada and Jarrosuelto, were evaluated. Their adaptability to two water stress regimes (moderate/deficit and severe/survival) was evaluated through the composition of intact aroma precursors compared with the predominant regional cultivar, Airén. The results revealed cultivar-dependent responses to water stress. Aroma precursor biosynthesis in Airén was only marginally affected, whereas Pintada exhibited enhanced accumulation under severe water deficit. Additionally, a C_13_-norisoprenoid hexose–hexose derivative and an isopropyl alcohol pentosyl-hexoside isomer were detected exclusively in Airén and Jarrosuelto, respectively, indicating their potential as varietal markers.

## Graphical abstract

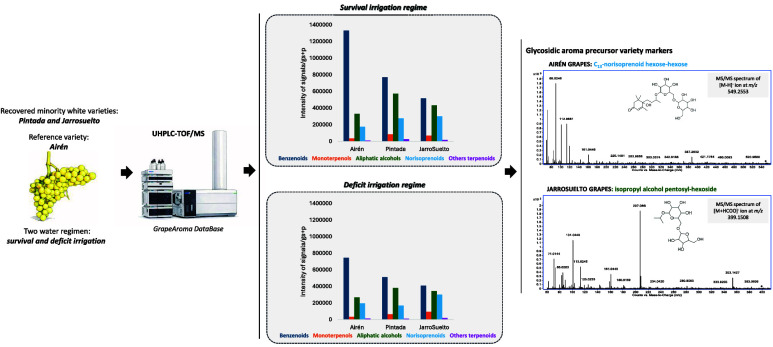

## Introduction

Climate change is redefining the soil and climate conditions of the main wine-growing regions, with increasingly significant effects on vine phenology, plant productivity, and grape compositional quality.^1,2^ The sustained increase in average temperatures, more frequent heat waves, and the reduction and greater irregularity of rainfall compromise water availability for the vine, which accelerates ripening and consequently alters the balance between primary (sugars and acids) and secondary (phenylpropanoids, terpenoids, norisoprenoids, among others) metabolites.^3^ This has an impact on the quality of the grapes and, consequently, on wine quality, as well as the regional typicity highly appreciated in many historically wine-growing areas. This scenario has prompted the search for different adaptation strategies, including field management practices, selection of more resistant rootstocks, and identification and recovery of local varieties with a predisposition to tolerate warm, water-deficient conditions, considering the wide genetic diversity available in wine-growing regions.^4–7^

Among the varietal diversification strategies, the recovery of native minority cultivars that have historically remained in marginal viticultural systems is emerging as an option of high agronomic and enological interest.^8–12^ In the Mediterranean region, some of these varieties, which have been selected over generations under Mediterranean-Continental and semiarid conditions, exhibit favorable phenotypic and adaptive characteristics in response to heat and drought stress, such as lower stomatal sensitivity.^13^ Castilla-La Mancha has been working on the recovery of native varieties for more than 20 years through the Institute of Vine and Wine of Castilla-La Mancha (IVICAM). Currently, these varieties are grown at the Grapevine Germplasm Bank of Castilla-La Mancha (GGBCM), which was created to preserve the high diversity of grape cultivars in this region. This Bank includes both red and white varieties, and, among the latter, Pintada and Jarrosuelto are particularly notable. To date, scientific research on these varieties remains incipient. Nevertheless, some studies have been conducted on the amino acid content in grape juice,^14^ the volatile compounds in wine,^12^ and the analysis of free volatiles and glycosidic precursors in grapes following acid^15^ and enzymatic hydrolysis.^16^ However, intact glycosidic aroma precursors in these recovered varieties (Pintada and Jarrosuelto) have not yet been investigated, despite their contribution to the sensory characteristics of wines. Moreover, investigating these compounds helps address a major limitation in the characterization of bound aroma compounds following acid or enzymatic hydrolysis. Establishing a direct relationship between glycoside composition and the resulting free aglycone profile is challenging, as acid hydrolysis can generate chemical products caused by intramolecular rearrangements of the released aglycones. Likewise, enzymatic hydrolysis may modify the resulting profile because variations in enzyme specificity can lead to different degrees of compound hydrolysis. As a result, the profiles obtained through these approaches may not faithfully reflect the composition of the original grapes.^17,18^ Although these techniques provide an estimate of glycoside-derived aroma compounds,^19^ the liberated aglycones are frequently sensorially inactive or correspond to odorless precursors that require slow chemical transformations to become aromatic. Therefore, correlating glycoside composition with free aglycone profiles after hydrolysis remains complex, particularly due to differences in glycosidase activity toward individual glycosides.^20^

The adaptation of recovered minority grape varieties to water stress not only determines their yield and phenology but also affects fruit composition. At the molecular and metabolic levels, water stress modulates the interplay between primary and secondary metabolism. Carbon and energy availability, derived from photosynthetic assimilation, influences the supply of substrates for the biosynthesis of terpenoids, phenylpropanoids, and norisoprenoids, among others.^21,22^ In parallel, hormonal signaling, mainly mediated by abscisic acid, and cellular redox status regulate the transcription of biosynthetic and conjugation genes.^21,23^ In this context, glycosylated compounds represent a critical fraction of the grape’s aroma reservoir.^24^ From a biosynthetic and genetic standpoint, their accumulation in the berry depends on both the aglycone biosynthesis (e.g., monoterpenes via the MEP pathway and norisoprenoids derived from carotenoid degradation) and the activity of glycosyltransferases (GTs) responsible for the conjugation of moieties to the aglycone.^22,25–28^ The characterization of the glycosidic volatile profile is functionally relevant for aroma release in wine and has practical applications in enology, including varietal traceability. Research on glycosidic compounds has demonstrated that they are variety-dependent and exhibit high relative stability, thereby serving as effective chemical markers for varietal discrimination, even in blended grapes. The resulting molecular signature can reliably distinguish among *Vitis vinifera* ^29^ varieties and represents a valuable tool for authenticity control and quality assurance.^30–32^ The application of advanced analytical techniques, such as ultrahigh-performance liquid chromatography coupled with quadrupole-time-of-flight mass spectrometry (UHPLC/QTOF), enables detailed profiling of glycosidic derivatives, including monoterpenes, norisoprenoids, and benzenoids. These profiles support their use in assessing the aroma potential and in variety identification of grape cultivars.^33–36^

Assessing the agronomic adaptation through analysis of the intact glycosidic volatile profile of recovered varieties can provide insight into their suitability under reduced water availability. This study focused on two recovered white grape varieties from Castilla-La Mancha, Pintada and Jarrosuelto, and compared them with the predominant white grape variety cultivated in the region, Airén. The research was conducted on vines subjected to two levels of water stress: survival (severe) and deficit (moderate). Accordingly, this study tests the hypothesis that adaptation to water stress influences the composition of intact volatile glycosides.

## Materials and Methods

### White Grapevines Varieties and Crop Conditions

Grapes from a widely cultivated variety (Airén, VIVC: 157) and two recovered varieties (Pintada, VIVC: 24142; Jarrosuelto, VIVC: 24138) were sampled in 2022 from a multivarietal experimental vineyard located at the Regional Institute of Agri-Food and Forestry Research and Development of Castilla-La Mancha (IRIAF), in Tomelloso, Castilla-La Mancha, Spain (latitude 39 °10′14″ N, longitude 3 °00′16′′ W; altitude 660 m. a.s.l.).

The experimental site and growing conditions have been previously described by Parra et al.^37^ The vineyard was established in 2008, grafted onto Fercal rootstock and trained using the VSP trellis system with bilateral Royat cordon pruning, leaving six two-bud spurs per vine. Cultivars were arranged in parallel rows, spaced 2.8 m apart, with 1.2 m between vines, resulting in a plating density of 2976 vines/ha. Each cultivar occupied a single row of 50 vines oriented at 30°NNE–210°SSW.

The climate of the area is semiarid Mediterranean with continental influence, with a marked thermal temperature amplitude of 21.5 °C. The average annual temperature is 14.8 °C, and the reference evapotranspiration (ETo) reaches 1290 mm. According to data from the Argamasilla de Alba weather station, part of the SIAR network operated by the Spanish Ministry of Agriculture, Fisheries, and Food (MAPA) and located 12 km from the experimental site, the average annual precipitation for the period 2011–2021 was 359 mm, while total precipitation in the 2022 growing season was 345 mm. Typically, only about 40% of the annual rainfall occurs during the grapevine growing season. The vineyard soil is classified as Petric Calcisol (FAO soil classification) or Petrocalcic Calcixerept (USDA soil classification), with a loam texture, active limestone, and organic matter contents of 15% and 3.2%, respectively. Soil depth is limited to 30 cm, below which a petrocalcic horizon restricts vine root penetration. This type of soil, widely distributed in the La Mancha wine region, is traditionally associated with grapevine cultivation. Overall, the pedoclimatic conditions correspond to a xeric moisture regime typical of Mediterranean climates.

### Grapevines Water Stress Conditions and Sampling

The experimental vineyard was managed under two irrigation regimes designed to impose contrasting levels of water stress, as previously described by Parra et al.^37^ The treatments were: (a) Severe water stress (SI regime), in which vines received only survival irrigation, and (b) Deficit water stress (DI regime), aimed at maintaining vines under moderate water deficit conditions. These regimes were confirmed using must carbon isotope composition (δ^13^C), with SI characterized by δ^13^C values > −24‰ and DI by −25‰ < δ^13^C < −24‰. δ^13^C parameter is used to determine the water status of the grapevine, since δ^13^C values of the grape must can be used to reflect how the vine manages water during fruit ripening. During drought, the plant is less efficient at absorbing ^13^C due to stomatal closure, which increases its proportion in the tissues. Therefore, δ^13^C serves as an integrated indicator of water stress in the grapevine.^38^

Irrigation requirements were determined according to Allen et al.,^39^ using a soil water balance approach based reference evapotranspiration (ET_o_) and effective precipitation measured at a meteorological station situated within the experimental vineyard. Irrigation was derived from reference evapotranspiration (ET_o_) adjusted with crop coefficient (*K*_c_) to estimate crop evapotranspiration (ET_c_). Irrigation water corresponded to a fraction of ET_c_, equivalent to 20% of ET_o_. Under the SI regime, this fraction was applied every 3–4 weeks, replacing only the ET_c_ corresponding to the week preceding each irrigation event. In contrast, under the DI regime, the same fraction of ET_c_ was applied weekly, replenishing the ET_c_ corresponding to the previous week to maintain moderate water deficit conditions. Irrigation was applied using a surface drip system equipped with self-compensating emitters (2.2 L/h), spaced at 0.6 m along the vine rows, with an assumed application efficiency of 95%. Irrigation events were scheduled at night to minimize evaporative losses. The irrigation season extended from the second week of June to the second week of September. Under the SI regime, water was applied every three to 4 weeks, only when visible symptoms of water stress (e.g., leaf wilting or defoliation) appeared. Under the DI regime, irrigation was applied weekly. The cumulative irrigation volumes supplied under each regime have been previously reported by Parra et al.^37^ Overall, vines under SI received 273 m^3^/ha, whereas vines under DI received 977 m^3^/ha of net irrigation water.

For each variety (Airén, Pintada, and Jarrosuelto), 50 vines were monitored. Of these, 20 vines were subjected to SI regime and 30 vines to the DI regime. To determine harvest date, berries were sampled periodically until total soluble solids between 20 and 22°Brix. At harvest, representative grape samples of approximately 1.5 kg per irrigation treatment were collected and immediately frozen for subsequent analysis.

### Sample Preparation for Glycosidic Aroma Precursors Analysis

The glycosidic aroma precursors were analyzed according to Cebrián-Tarancón research.^32^ Twenty-five berries were randomly collected, weighed, and manually separated into pulp and skins (seeds removed). The skins were extracted by immersion in 35 mL of methanol for 4 h, followed by homogenization using an Ultra-Turrax homogenizer (IKA Werke, Staufen, Germany) and centrifugation at 4000 rpm for 10 min. Methanol was removed under reduced pressure using a rotary vacuum evaporator (LABOROTA 4000eco; Heidolph Instruments, Schwabach, Germany), and the extract was concentrated to a final volume of 10 mL at 40 °C. The volume was then adjusted to 100 mL with water. Polyclar AT (1 g) was added to remove polyphenols and tannins, and the mixture was stirred for 20 min and then centrifuged at 4000 rpm for 10 min. The supernatant was recovered. The pulp fraction was treated with 50 mg of sodium metabisulfite and centrifuged at 4000 rpm for 10 min. The supernatant was collected, adjusted to 100 mL with water, and supplemented with 40 mg of a pectolytic enzyme (Pectazina DC, Dal Cin, Concorezzo, MB, Italy). The mixture was incubated at room temperature for 4 h and subsequently centrifuged. Equal aliquots (10 mL) of skin and pulp extracts were combined, and 200 μL of a methanolic *n*-heptyl glucoside solution (40 mg/L) was added as an internal standard. The extract was loaded onto a 1 g Sep-Pak C18 cartridge (Waters Corporation, Milford, MA, USA) previously conditioned with dichloromethane, methanol, and water. Free volatile compounds were eluted with 5 mL of dichloromethane, whereas glycosylated aroma compounds were eluted with 5 mL of methanol. The eluates were filtered through a 0.22 μm PTFE membrane (Waters Corporation, Milford, MA, USA) and collected in vials for LC–MS analysis. For each sample, three independent grape extracts (biological replicates) were prepared and analyzed.

### UHPLC/QTOF Mass Spectrometry

Analyses were performed in negative ionization mode using an ultrahigh-performance liquid chromatography (UHPLC) Agilent 1290 Infinity system coupled to an Agilent 1290 Infinity autosampler (G4226A) and an Agilent 6550 iFunnel accurate-mass Quadrupole Time-Of-Flight (QTOF) Mass Spectrometer (40,000 resolving power fwhm) equipped with Dual Agilent Jet Stream Ionization source (Agilent Technologies, Santa Clara, CA).

Chromatographic conditions were previously described by Cebrián-Tarancón research.^32^ The chromatographic separation was performed using a Zorbax reverse-phase column (RRHD SB-C18, 3 mm × 150 mm, 1.8 μm; Agilent Technologies, Santa Clara, CA). The mobile phase consisted of (A) 0.1% (v/v) aqueous formic acid and (B) 0.1% (v/v) formic acid in acetonitrile. The gradient elution program was as follows: 5% B held isocratically for 8 min; 5 to 45% B over 10 min; 45 to 65% B over 5 min; 65 to 90% B over 4 min; and 90% B held for 10 min. The flow rate was set at 0.4 mL/min, the injection volume was 5 μL, and the column temperature was maintained at 35 °C. The absence of carryover and false positives was verified by injecting a blank consisting of mobile phases A/B (1:1, v/v) after each sample.

QTOF-MS analyses were performed under the following conditions: sheath gas (N_2_) at 12 L/min and 350 °C, drying gas at 16 L/min and 250 °C, nebulizer pressure at 40 psi, nozzle voltage set to 0 kV (negative ion mode), and capillary voltage set to −3.5 kV. Data were acquired over an *m*/*z* range of 100–1700 at an acquisition rate of 2 spectra/s. Mass calibration was carried out using a standard reference mixture (G1969–85000, Supelco Inc.), achieving a residual mass error of ±0.2 ppm. Lock mass correction in negative ionization mode was performed using the trifluoroacetic acid (TFA) anion at *m*/*z* 112.9856 and HP-0921 at *m*/*z* 966.0007 (ion [M + HCOO]^−^).

Glycosidic volatile precursors were identified into the in-house database of grape glycosidic aroma compounds (*GrapeAroma*) using Agilent MassHunter Qualitative Analysis software (B.06.00, 6.0.633.0) and the “*Find by Formula*” algorithm and confirmed by performing MS/MS experiments. The database was previously described.^31^ Briefly, it includes names and formulas of grape glycosidic benzenoids, monoterpenes, and norisoprenoids identified in successive studies. Compounds were annotated with their common names (e.g., linalool pentosyl-hexoside) or provide generic information regarding the aglycone backbone and glycosidic moiety (e.g., C_13_-norisoprenoid hexose). Compounds having the same molecular formula and one or more substituents sited in different positions on the molecule, or differing in stereochemistry, were eluted at different retention times in the chromatogram. They could not distinguish by HR-MS/MS therefore they were annotated as isomers. Currently, the database consists of 71 entries.

The overall identification score was calculated as a weighted average of isotopic pattern parameters, including exact mass, relative abundance, and *m*/*z* spacing, using weighting factors of 100, 60, and 50, respectively. Tolerances were set to 2.0 mDa + 5.6 ppm for mass accuracy, 7.5% for isotope abundance, and 0.0025 *m*/*z* + 7.0 ppm for isotope spacing.

MS/MS analyses were performed by selecting precursor ions in the *m*/*z* range of 100–1700 and applying collision energies between 20 and 60 eV, with an acquisition rate of 2 spectra/s. For each metabolite, the intensities of the [M + HCOO]^−^ and [M – H]^−^ ions were normalized to the corresponding signal of the internal standard (*n*-heptyl glucoside), and the sum of the two normalized intensities was calculated. Metabolite identification was assigned according to the Metabolomics Standards Initiative guidelines.^40^ For compounds not included in the GrapeAroma database, MS/MS spectra were compared with in-silico fragmentation patterns of the proposed structures using Molecular Structure Correlator (MSC) software, and identification confidence was expressed as a percentage match score.

### Statistical Analyses

Two-way ANOVA was conducted to determine the statistical significance of cultivar (V), water stress regime (W), and their interaction (VxW) in the values of compounds analyzed. Detailed analysis of glycosidic volatile precursors was carried out using a *t*-test at a 95% probability level to determine differences between the widespread variety (Airén) and the two recovered cultivars (Pintada and Jarrosuelto), as well as among water stress regimes within each variety. Finally, a principal component analysis (PCA) was performed to obtain an assessment of the influence of the variety and water stress regime on the accumulation of glycosidic volatile precursors. The statistical software package used was Statgraphics Centurion statistical program (version 19.4.02; StatPoint, Inc., The Plains, VA, USA).

## Results and Discussion

### Glycosydic Aroma Precursors of Airen, Pintada, and Jarrosuelto

The putative intact glycosylated compounds identified in Airén, Pintada, and Jarrosuelto grapes are summarized in Table S1. A total of 20 compounds were detected, belonging to the chemical classes of benzenoids, monoterpenols, aliphatic alcohols, and norisoprenoids including hexoside and pentosyl-hexoside derivatives. These compounds have recently been reported in the two recovered Spanish minority red grape varieties Moravia Agria and Tinto Fragoso, and in Tempranillo grapes.^32^

Due to the lack of commercially available standards for the identified metabolites, a comparative study among the metabolites using a relative quantification has been carried out. A comparative among Airén, Pintada, and Jarrosuelto grape samples was performed using the [M – H]^−^ ion signal intensities of compounds detected in the combined skin and pulp extracts, normalized to that of the internal standard (Table S2). This method has been successfully applied in previous studies on grape samples from different varieties or subjected to different withering processes.^32,35,36^

### Effects of Variety, Water Stress and Their Interaction in Glycosidic Aroma Precursors

The effects of “*variety*” (V) and “*water-stress* regime” (W) were evaluated using two-way ANOVA with normalized signal intensities of the identified glycosidic metabolites as variables. The results are summarized in Table 1. Considering the two factors separately, “*variety*” (V) exerted a greater effect on the metabolite profile, except for isopropyl alcohol pentosyl-hexoside isomer 1; 3-methyl-2-buten-1-ol pentosyl-hexoside, 1-hexanol pentosyl-hexoside isomer 1, and geranic acid pentosyl-hexoside isomer 2. In particular, the signal of monoterpendiol pentosyl-hexoside stood out from the others, showing a high level of significance. Conversely, the “*water stress regime”* (W) factor affected only a limited number of metabolites, such as dimethoxyphenol hexoside; monoterpendiol pentosyl-hexoside; 3-methyl-2-buten-1-ol pentosyl-hexoside; geranic acid pentosyl-hexoside isomer 1; and geranic acid rhamnosyl-hexoside. Analysis of the two-way interaction (“*variety* × *water stress regime*”) revealed a significant effect for most compounds, except benzyl alcohol pentosyl-hexoside; hydroxylinalool/furanlinalooloxide hexoside; geraniol pentosyl-hexoside; isopropyl-alcohol pentosyl-hexoside isomer 1; 3-hexen-1-ol hexosyl-hexoside; and C_13_-norisoprenoid-hexose isomer. Notably, the signals of geranic acid pentosyl-hexoside isomer 1, the two 1-hexanol pentosyl-hexoside isomers, 3-methyl-2-buten-1-ol pentosyl-hexoside, linalool pentosyl-hexoside, nerol pentosyl-hexoside, and dimethoxyphenol hexoside exhibited the highest statistical significance (*p* < 0.001, Table 1).

**Table 1:** Significance of the Variety (V), Water Regime (W) and Their Interaction on Values of Putative Glycosidic Aroma Precursors^a^

	variety (V)	water stress regime (W)	V × W
Benzenoids			
dimethoxyphenol hexoside	84.52***	38.52***	13.43***
benzyl alcohol pentosyl-hexoside	7.84**	4.24	1.45
Monoterpenols			
hydroxylinalool/furanlinalooloxide hexoside isomer	61.22***	0.03	1.07
monoterpendiol pentosyl-hexoside	169.39***	5.92*	10.53**
linalool pentosyl-hexoside	98.10***	1.48	63.00***
nerol pentosyl-hexoside	47.77***	3.57	16.36***
geraniol pentosyl-hexoside	17.43***	1.60	0.51
Aliphatic Alcohols			
isopropyl-alcohol pentosyl-hexoside isomer 1	1.24	0.35	1.83
2-butanol pentosyl-hexoside	35.91***	2.92	4.52*
3-methyl-2-buten-1-ol pentosyl-hexoside	3.01	26.69***	20.23***
3-hexen-1-ol hexosyl-hexoside	10.92**	2.64	2.37
1-hexanol pentosyl-hexoside isomer 1	2.71	1.94	16.82***
1-hexanol pentosyl-hexoside isomer 2	57.56***	4.37	16.31***
Norisoprenoids			
C_13_–norisoprenoid-hexose isomer	9.77**	2.81	0.11
vomifoliol hexoside isomer	16.57***	1.73	4.55*
Other Terpenoids			
geranic acid pentosyl-hexoside isomer 1	77.09***	33.79***	81.10***
geranic acid rhamnosyl-hexoside	44.06***	14.53**	6.34*
geranic acid pentosyl-hexoside isomer 2	3.62	0.02	6.49*

a ANOVA test significance level: *p value <0.05, **p value <0.01, ***p value <0.001 and typed in bold.

The results suggest that variation in aroma precursors in grapes is primarily influenced by grape variety, rather than the level of drought imposed. This conclusion aligns with findings from previous studies examining both white and red grape varieties, which indicated that the levels of glycosidic aroma precursors and their aglycones were more strongly affected by the grape variety than by water stress levels.^22,28,37,41^ Nevertheless, when considering the simultaneous influence of both factors, a significant V × W interaction was found for some compounds. This indicates that different varieties exhibit distinct responses to water availability, suggesting that some may be more resilient to water stress than others. Such behavior has been previously observed in two Spanish recovered red grape varieties, Tinto Fragoso and Moravia, as well as in Tempranillo, a widely cultivated red grape variety.^32^ The findings of this study are consistent with prior research on the impact of water availability on the regulation of specific glycosyltransferases involved in the modulation of secondary metabolites in grapes, particularly volatile compounds.^22,42^ Specifically, deficit irrigation has been recognized as a common viticultural practice aimed at enhancing the concentration of glycosidic aroma precursors in grapes. However, this effect depends on factors such as grape variety, phenological stage at which water deficit occurs, and the geographic region under study.^43,44^

### Glycosidic Aroma Precursors: Family Responses

To compare the water-stress adaptation of the Pintada and Jarrosuelto varieties relative to the traditional Airén variety, a *t* test analysis was conducted using the normalized signal intensities of metabolites expressed per gram of grape. As no commercial standards are available for the quantification of these compounds, the data are represented as relative quantification. The results presented in Table 2 show the comparison between Pintada and Jarrosuelto and Airén under identical water stress conditions (indicated by small and capital letters), as well as the differences observed within each variety under two distinct water supply conditions (denoted by asterisks on the right). The results revealed several statistically significant differences associated with irrigation regime.

**Table 2:** Effect of Variety and Water Regime on Putative Glycosidic Aroma Precursors^a^

	survival irrigation regime			deficit irrigation regime			*p* value		
	Airén	Pintada	Jarrosuelto	Airén	Pintada	Jarrosuelto	Airén	Pintada	Jarrosuelto
Benzenoids									
dimethoxyphenol hexoside	1,176,038 ± 206255b,B	500,480 ± 53,383a	228,522 ± 6250A	588,621 ± 61,919b,B	334,639 ± 22,004a	167,306 ± 47,576A	**	***	
benzyl alcohol pentosyl-hexoside	155,696 ± 11,114a,A	270,991 ± 20,188b	289,900 ± 94,077A	156,927 ± 28,180a,A	177,130 ± 29,719a	241,741 ± 54,399A		*	
*total benzenoids*	*1,331,734 ± 199,102b,B*	*771,470 ± 63,006a*	*518,422 ± 92,552A*	*745,548 ± 61,105b,B*	*511,769 ± 51,712a*	*409,047 ± 91,002A*	**	**	
Monoterpenols									
hydroxylinalool/furanlinalool oxide hexoside isomer	7072 ± 1368a,A	7582 ± 611a	38,723 ± 13,732B	3377 ± 142a,A	6092 ± 198b	45,504 ± 8046B	**	*	
monoterpendiol pentosyl-hexoside	20,490 ± 2912a,B	51,500 ± 3448b	5969 ± 676A	21,734 ± 4808a,B	36,592 ± 5491b	7441 ± 1134A		*	
linalool pentosyl-hexoside	1522 ± 379a,A	4236 ± 358b	2986 ± 204B	1131 ± 216a,A	2259 ± 618b	6037 ± 449B		**	***
nerol pentosyl-hexoside	3910 ± 316a,A	13,199 ± 848b	11,313 ± 1127B	3229 ± 516a,A	9316 ± 2080b	22,139 ± 5123B		*	*
geraniol pentosyl-hexoside	3277 ± 755a, A	8758 ± 1416b	10,328 ± 210B	3229 ± 516a,A	10,514 ± 2833b	13,294 ± 5473B			
*total monoterpenols*	*36,271 ± 3535a,A*	*85,275 ± 5742b*	*69,319 ± 15,411B*	*32,699 ± 4017a,A*	*64,773 ± 10417b*	*94,416 ± 8243B*		*	
Aliphatic Alcohols									
isopropyl alcohol pentosyl-hexoside isomer 1	19,749 ± 58,44a,A	12,563 ± 1938a	15,107 ± 5664A	13,889 ± 1925a,A	14,294 ± 922a	16,064 ± 3347A			
2-butanol pentosyl-hexoside	47,381 ± 7323b,B	25,427 ± 479a	17,167 ± 1458A	37,352 ± 4189b,A	17,015 ± 4639a	23,258 ± 7864A		*	
3-methyl-2-buten-1-ol pentosyl-hexoside	88,684 ± 11,655a,A	110,878 ± 5241b	141,159 ± 43,860A	95,920 ± 22,534a,A	88,539 ± 12,223a	n.d.		*	**
3-hexen-1-ol hexosyl-hexoside isomer	151,856 ± 22,690a,A	402,396 ± 8072b	242,717 ± 150,249A	110,209 ± 24,396a,A	248,402 ± 61,404b	270,548 ± 67,054B		*	
1-hexanol pentosyl-hexoside isomer 1	17,019 ± 78a,B	14,598 ± 7042a	9082 ± 1793A	4606 ± 258a,A	6679 ± 2079a	21,604 ± 6110B	***		*
1-hexanol pentosyl-hexoside isomer 2	6379 ± 672a,A	6154 ± 190a	8494 ± 737B	5501 ± 389a,A	5400 ± 976a	12,753 ± 1614B			*
*total aliphatic alcohols*	*331,067 ± 28,499a,A*	*572,016 ± 6508b*	*433,725 ± 192,411A*	*267,476 ± 44,708a,A*	*380,329 ± 77,440a*	*344,228 ± 78906A*		*	
Norisoprenoids									
C_13_–Norisoprenoid hexose isomer	46,385 ± 4074b,A	31,613 ± 1320a	35,172 ± 9789A	42,006 ± 5162b,A	24,487 ± 4602a	31,359 ± 9387A			
vomifoliol hexoside isomer	130,324 ± 6833a,A	244,531 ± 27877b	265,435 ± 53,617B	153,529 ± 23,795a,A	144,760 ± 31,197a	270,978 ± 59,164B		*	
*total norisoprenoids*	*176,710 ± 10,285a,A*	*276,144 ± 28,646b*	*300,607 ± 62,986B*	*195,534 ± 25,166a,A*	*169,248 ± 35,573a*	*302,337 ± 64,671B*		*	
Other Terpenoids									
geranic acid pentosyl-hexoside isomer 1	n.d.	7728 ± 401a	5616 ± 319A	1704 ± 417a,A	n.d.	5943 ± 1563B	**	***	
geranic acid rhamnosyl-hexoside	4533 ± 217a,A	10,907 ± 869b	8162 ± 879B	3576 ± 341a,A	6949 ± 1332b	7914 ± 1427B	*	*	
geranic acid pentosyl-hexoside isomer 2	5524 ± 673a,B	7350 ± 428b	3934 ± 361A	7643 ± 2182a,A	4272 ± 697a	5133 ± 2156A		**	
*total other terpenoids*	*10,058 ± 528a,A*	*25,985 ± 904b*	*17,712 ± 1523B*	*12,923 ± 2548a,A*	*11221 ± 2029a*	*18,990 ± 3017A*		***	
*total glycosydic aroma precursors*	*1,885,840 ± 199315a,A*	*1,730,890 ± 83,128a*	*1,339,785 ± 349,097A*	*1,254,181 ± 113,991a,A*	*1,137,340 ± 148,648a*	*1,169,018 ± 151,820A*	**	**	

a The mean values (*n* = 3) are shown with their standard deviation. For each compound and irrigation regime, small letters indicate significant differences between Airén and Pintada varieties and capital letters indicate significant differences between Airén and Jarrosuelto varieties according to *t* test (*p* value <0.05). Also, different asterisks (*p value <0.05; **p value <0.01; ***p value <0.001) indicate differences between irrigation regime for each variety according to *t* test. n.d.: not detected.

#### Benzenoids

Benzenoids exhibited clear cultivar-dependent differences under both irrigation regimes. Airén showed significantly higher signals of dimethoxyphenol hexoside and total benzenoids compared with Pintada and Jarrosuelto, particularly under survival irrigation. In contrast, benzyl alcohol pentosyl-hexoside tended to be higher in the recovered varieties, especially in Jarrosuelto (Table 2). Generally, dimethoxyphenols are linked to organoleptic characteristics including spicy, smoky, and phenolic notes.^45^ Furthermore, the accumulation of their glycosidic derivatives in grapes has been directly linked to the occurrence of bushfires in proximity to wine-growing areas.^46^ While the metabolic pathways involved in benzenoid biosynthesis are not fully understood, it is established that benzyl alcohol is synthesized in plants via the phenylpropanoid pathway, catalyzed by the enzyme phenylalanine ammonia-lyase (PAL).^47^ PAL catalyzes the initial step in phenylpropanoid metabolism, converting phenylalanine into *trans*-cinnamic acid,^48^ which is subsequently transformed into benzyl alcohol and its derivatives. Savoi et al. demonstrated that grapevine responds to drought stress by modulating several secondary metabolic pathways, particularly through stimulation of of phenylpropanoids and volatile organic compounds.^27^ The results obtained in the present study suggest that, in these varieties, especially Pintada, reduced water supply increases oxidative stress levels, thereby activating gene expression and PAL activity. Previous studies have documented the quantification of benzyl alcohol in Airén wines^49^ and have indicated that these levels are lower in Pintada and Jarrosuelto grapes in comparison to Airén.^15^ According to Díaz-Fernandez et al.^12^ and compared with 27 other Spanish minority varieties, Jarrosuelto wines exhibited one of the highest concentrations of benzyl alcohol, reaching up to 5.03 mg/L. This study suggested that the presence of benzyl alcohol in wine is primarily attributed to varietal origin. In grapes, this compound can exist in both free and bound forms,^50,51^ and its glycosylated derivatives have been identified in several varieties.^31–33,36^ The release of aglycones in wine during fermentation may account for the heightened aromatic perception noted in the minority wines analyzed by Díaz-Fernández et al.^12^ Various descriptors for benzyl alcohol are present in the literature, including sweet and fruity,^52,53^ spicy and balsamic,^54^ and floral.^50^ Nonetheless, benzyl alcohol glycosides are considered to have minimal impact on wine aroma.^19^

#### Monoterpenols

The five monoterpenol glycosides identified in the samples, including four pentosyl-hexoside and one hexoside derivatives (Table 2), had clear cultivar-dependent differences under both irrigation regimes. Pintada and Jarrosuelto showed significantly higher concentrations of total of monoterpenols compared with Airén, mainly due to linalool, nerol, and geraniol pentosyl-hexosides, as well as hydroxylinalool/furanlinalool oxide hexoside. Notably, the concentration of the latter compound was approximately an order of magnitude higher in the Jarrosuelto samples. Overall, both varieties exhibited similar patterns, with increased monoterpenol accumulation noted under SI conditions. Conversely, irrigation influenced Jarrosuelto differently, as the synthesis of linalool and nerol pentosyl-hexosides was enhanced under moderate water stress conditions (DI).

Among the identified monoterpenes, the highest signal detected corresponds to a monoterpenoid pentosyl-hexoside with the molecular formula C_21_H_36_O_11_, which eluted at 15.28 min in the chromatogram. In addition, the specific aglycone remains unidentified. However, the monoterpenol glycosides identified in these varieties have been previously characterized in other white grape varieties as well as in the red grapes cultivars Moravia Agria and Tinto Fragoso.^31,32,35^ Monoterpenes constitute the predominant class of grape aroma compounds, with linalool, nerol, and geraniol being the most significant.^55^ Their important contribution to the wine aroma is due to the low sensory thresholds and their interactions with other compounds.^56^ These compounds confer sensory notes such as orange flowers and lavender (linalool), floral and orange flowers (nerol), geranium and rose (geraniol).^45,57,58^ Previous studies have identified both free and bound linalool and geraniol in Jarrosuelto grapes.^16^ Notably, higher levels of geraniol were observed in the volatile profiles of Pintada and Jarrosuelto when compared with Airén, while the linalool concentrations across the three varieties were found to be similar.^15^ However, these findings align with the present results, although the previous investigation involved the analysis of aglycones following acid hydrolysis, a process that may have introduced artifacts.^20^ According to Díaz-Fernández et al.^12^ the wines produced from Jarrosuelto grapes contained significant concentrations of linalool, nerol, and geraniol. 8-Hydroxylinalool was not found in the volatile profiles of grapes and wines from these varieties.^12,15,16,59^ This compound has an odor threshold higher than linalool and is characterized by a citrus aroma, a slightly sweet character, with soapy or floral notes.^60^

The clear cultivar-dependent differences in monoterpenols are supported by the literature since previous studies have documented an increase in grape monoterpenes under light to moderate water stress conditions, including limonene, linalool, α-terpineol, geranyl acetone, geraniol, and citronellol.^27,43^ In Chardonnay, this increase has been linked to the heightened expression of terpenoid synthase genes.^25^

In *Vitis vinifera*, monoterpene synthesis occurs mainly through the plastidial MEP pathway (2-C-methyl-d-erythritol-4-phosphate or DOXP), involving key enzymes such as DXS, DXR, GPPS, and terpene synthases, which catalyze the formation of C_10_ aroma compounds. In white grape varieties, several studies have shown that water deficit alters the expression of MEP pathway genes leading to changes in both free and glycosylated monoterpene accumulation. It is known that these variations depend on the cultivar and the timing of stress. However, hormones such as abscisic acid and other signaling molecules, including jasmonates and ethylene, along with drought-responsive transcription factors, regulate this pathway, influencing aroma composition. Thus, drought stress intensity and timing (before or after veraison) play a critical role in determining the aromatic composition of white grapes under water stress.^21,27,61^

#### Aliphatic Alcohols

In the samples studied, six aliphatic alcohols glycosides were identified (Table 1). For these compounds, the signal intensities varied depending on the grape cultivar (Table 2). Pintada showed significantly higher concentrations of 3-methyl-2-buten-1-ol pentosyl-hexoside, 3-hexen-1-ol hexosyl-hexoside isomer and total aliphatic alcohols compared with Airén, particularly under survival irrigation. In contrast, 2-butanol pentosyl-hexoside and 1-hexanol pentosyl-hexoside isomer 1 tended to be higher in Airén variety under the same water regimen (Table 2). In Jarrosuelto, the signals of 1-hexanol pentosyl-hexoside isomers and 3-hexen-1-ol hexosyl-hexoside isomer were higher than in Airén grapes, especially under deficit irrigation. All of these aliphatic alcohols have been previously found in other white grapes varieties^31,33,36^ and in the red cultivars Moravia Agria and Tinto Fragoso.^32^ When the free forms of these compounds were analyzed, the analysis revealed a lower content of 1-hexanol in Airén grapes compared with Pintada and Jarrosuelto, while the highest concentration of 3-hexen-1-ol was detected in Airén. Similar findings have been reported in grapes from these varieties subjected to rainfed and irrigated conditions.^15^ In the context of wines, Jarrosuelto exhibited lower concentrations of these volatile compounds compared with other Spanish minority white wines.^12^ From the point of view of sensory impact, aliphatic alcohols are primarily associated with negative sensory attributes in wine, such as alcoholic (2-butanol), chemical/medicinal (isopropyl alcohol), green/herbaceous (3-methyl-2-buten-1-ol, 3-hexen-1-ol), cream/resinous (1-hexanol).^19,45,58^ The results observed for 1-hexanol pentosyl-hexoside isomer signals in Jarrosuelto grapes under moderate water deficit are consistent with observations reported in Moravia Agria and Tinto Fragoso grape varieties.^32^ On the other hand, this finding contradicts a prior study that reported that water deficit decreases the content of aliphatic alcohols.^62^

C_6_-compounds are primarily formed through the lipoxygenase–hydroperoxide lyase pathway (LOX–HPL), which acts on polyunsaturated fatty acids. In this process, the LOX enzyme oxidizes fatty acids to generate hydroperoxides, which are then cleaved by HPL to produce C_6_ aldehydes and then are converted by alcohol dehydrogenase in C_6_-alcohols.^25,63^ Several studies have shown that under water deficit the expression of LOX and HPL genes increases, thereby promoting the synthesis of these compounds. However, some authors have noted that the response may be influenced by the grape variety and the developmental stage of the grape.^64,65^

#### Norisoprenoids

In the samples, three norisoprenoid compounds were identified (Table S1). Consistent with earlier studies, HR-MS/MS predominantly yielded the ions at *m*/*z* 223.1349 and 191.0557, which lead to identification of putative vomifoliol-hexose, at *m*/*z* 387.2013 and 225.1481 indicative of a putative C_13_-norisoprenoid hexose–hexose derivative, and at *m*/*z* 227.1636 and 155.1074 consistent with a C_13_-norisoprenoid hexose. Formation of fragment ions resulting from the loss of sugar moieties was observed across all compounds, thereby confirming their glycosidic character. However, a definitive identification of the aglycone backbone was not achievable, leading to their classification merely as glycosidic C_13_-norisoprenoids.

Along with these findings, norisoprenoids exhibited clear cultivar-dependent differences under both irrigation regimes. Both minority varieties showed higher signals of vomifoliol hexoside isomer and total norisoprenoids compared with Airén, which were particularly significant under survival irrigation (Table 2). Previous studies have identified these norisoprenoids in other white grape varieties^31–33,36^ and quantified their aglycones in Jarrosuelto grapes.^16^ The odor descriptors of these compounds include floral and fruity notes, possessing low sensory thresholds that can significantly influence wine aroma.^66^ C_13_-norisoprenoids are produced through the oxidative degradation of carotenoids found in the berry skin and pulp.^66^ This process is facilitated by carotenoid-cleavage dioxygenases (CCDs), specifically the enzymes VvCCD1 and VvCCD4, which are essential for converting carotenoids into shorter molecules known as apocarotenoids.^67^ These intermediates then undergo further enzymatic and chemical reactions, including oxidation and isomerization, culminating in the formation of C_13_-norisoprenoids. Research indicates that moderate water stress can enhance the formation of these compounds, thereby increasing the aroma potential of white grapes.^27^

#### Other Terpenoids

The compounds classified under the category of other terpenoids (Table 2) revealed significant cultivar-dependent differences under both irrigation regimes. Pintada and Jarrosuelto showed significantly higher signals of geranic acid rhamnosyl-hexoside compared with Airén for both irrigation regimes. However, these significant differences were observed for the total other terpenoids only under survival irrigation (Table 2). These geranic acid derivatives have been previously reported in the grapes of Moravia Agria, Tinto Fragoso, and Tempranillo,^32^ as well as in those of Chardonnay, Muscat Blanc, and Riesling.^33^ The odor descriptor of geranic acid is associated with green notes^45^ and the presence of this compound had not previously been reported in grapes and wines from Spanish recovered grape varieties,^12,15,16^ but it was quantified in the Airén grape during the ripening process.^59^ The lack of identification in earlier studies may be attributed to the fact that under enological conditions, its structure can undergo acid-catalyzed isomerization, oxidation, and esterification. Additionally, enzymatic and oxidative processes occurring within grape tissues and during fermentation can rapidly convert geranic acid into other monoterpenoids, such as linalool, α-terpineol, or geranyl acetate.^30,68^

The findings from this research suggest that Pintada is a variety particularly sensitive to water stress, leading to a heightened activation of metabolic pathways involved in glycoside synthesis. In contrast, in Airén and Jarrosuelto signal intensities remained relatively stable for almost all the glycosidic metabolites under water-deficit conditions, resulting in minimal variation in the profile of glycosidic aroma precursors (Table 2).

### Effect of Water Management on Grape Glycosidic Aroma Precursors

As has been indicated for each family of compounds, the synthesis of their free forms is affected by water availability. However, variations in their levels depend not only on the modulation of their primary biosynthetic pathways, but also on the activity of the glycosylation pathways that determine whether they occur in the free or bound forms. Glycosylation, which is mediated by glycosyltransferase enzymes, regulates the stability, solubility, and storage of these metabolites in plant tissues. In this context, water deficit has been shown to induce the expression of genes involved in the glycosylation of volatile metabolites. For instance, in Sangiovese grape berries, a significant upregulation of specific glycosyltransferases associated with monoterpene glycosylation has been reported under severe preveraison water stress conditions.^28^ It is known that, under water stress conditions, the grapevine increases the synthesis of abscisic acid, a key hormone in stomatal regulation and response to drought, to limit water loss and activate tolerance mechanisms.^69^ Simultaneously, abscisic acid can induce the expression of glycosyltransferases in grapes.^21^ The glycosylation process allows these compounds to be stored in a nonvolatile and stable form, protecting them from oxidative degradation during water stress conditions.^33^ Furthermore, recent studies have demonstrated the importance of abscisic acid to regulate the expression of glycosylation-related genes in grapes. Under water stress conditions, abscisic acid biosynthesis in roots increases, as does its transport to other parts of the plant, regulating the expression these genes.^70^

The physiological response of grapevine varieties to abiotic conditions is strongly cultivar-dependent, reflecting inherent differences in stomatal regulation, hydraulic architecture, and metabolic strategies. These varietal differences determine the plant’s sensitivity and adaptive responses to changes in water potential, influencing transpiration, photosynthesis, and secondary metabolite accumulation.^71^ In this context, Serrano et al. classified recovered grapevine varieties from Castilla-La Mancha region in comparison with national and international varieties based on their responses to water potential.^7^ The results showed that Airén variety was identified as extremely anisohydric, meaning that it maintained this behavior even under severe water stress. This indicates that it keeps its stomata open for photosynthesis despite limited water availability, though this may increase its susceptibility to water stress. For its part, Jarrosuelto was also classified as anisohydric, but only up to weak-mild stress. However, Pintada was categorized as partially isohydric, meaning it regulates its water potential more strictly, closing its stomata during drought to prevent water loss. This response protects it from water stress but also limits photosynthesis and growth during drought periods. This classification underscores the cultivar-specific hydraulic strategies, indicating that such variations in water-use behavior are likely to modulate the biosynthesis and accumulation of glycosylated aroma precursors. In isohydric varieties, the water deficit signal activates the synthesis and transport of abscisic acid from the roots to the leaves, where it accumulates, promoting stomatal closure to prevent excessive water loss through transpiration. This stomatal closure has a direct effect on primary metabolism, especially photosynthesis, and leads to a decrease in stomatal conductance, O_2_ accumulation, and a drop in sugar production.^72,73^ Moreover, the stomatal closure observed in isohydric varieties may initially reduce the carbon flux available for sugar-dependent secondary metabolism, including the synthesis of glycosylated precursors. Consequently, secondary metabolism is therefore affected more indirectly by stomatal closure, which can be explained through two interrelated mechanisms: first, as previously mentioned, through an indirect impact resulting from the restriction of primary metabolism; second, through a direct effect triggered by abscisic acid and oxidative stress. In this context, abscisic acid acts not only as a signal mediating stomatal closure but also as a transcriptional regulator that activates secondary metabolism genes, such as glycosyltransferases, involved in the conjugation of volatile compounds.^25,26^ This mechanism would explain the greater accumulation of glycosylated aroma precursors in Pintada grapes compared to Jarrosuelto. It is true that in anisohydric varieties, such as Jarrosuelto, stomatal opening maintains the activity of primary metabolism, but secondary metabolism (including the glycosylation of volatile compounds such as monoterpenes, norisoprenoids, and benzenoids) can maintain a basal level or even decline compared to that of an isohydric variety exposed to the same stress, due to reduced abscisic acid signaling and lower oxidative stress. The same justification, but even more pronounced, could be applied to the Airén variety, classified as extremely anisohydric. If this behavior is compared with that shown by recovered red varieties (Tinto Fragoso and Moravia Agria) and Tempranillo, a contrasting pattern is observed: the anisohydric variety (Moravia Agria) was the one with the highest accumulation of glycosylated aroma precursors.^32^ This observation may be explained by the fact that the variety exhibiting the higher accumulation of glycosylated volatiles (Moravia Agria) had lower levels of phenolic compounds, particularly anthocyanins. This is consistent with evidence suggesting that enhanced synthesis of volatile glycosides can lead to a reduction in phenolic content, likely due to competition for shared metabolic precursors, resulting in a preferential allocation of resources to one pathway over the other.^74,75^ In the case of white varieties, obviously, the synthesis pathway of anthocyanins does not compete with the synthesis pathway of glycosylated volatile compounds. However, Parra et al. showed that the accumulation of phenolic compounds (phenolic acids, flavanols and flavonols) in Jarrosuelto grapes was greater than in Pintada under water stress conditions.^13^

### Variety Differentiation and Glycosidic Markers Identification

To reduce the dimensionality of the data set and identify a small number of linear combinations of the variables that captured most of the variability, principal component analysis (PCA) was performed using the normalized signal intensities of the aroma precursors listed in Table 2. The first two principal components were selected to illustrate the distribution of the samples in the multivariate space (Figure 1). The first component explained 42.82% of the variability, while the second component accounted for 17.02%, together representing 60% of the total of variance.

**Figure 1:**
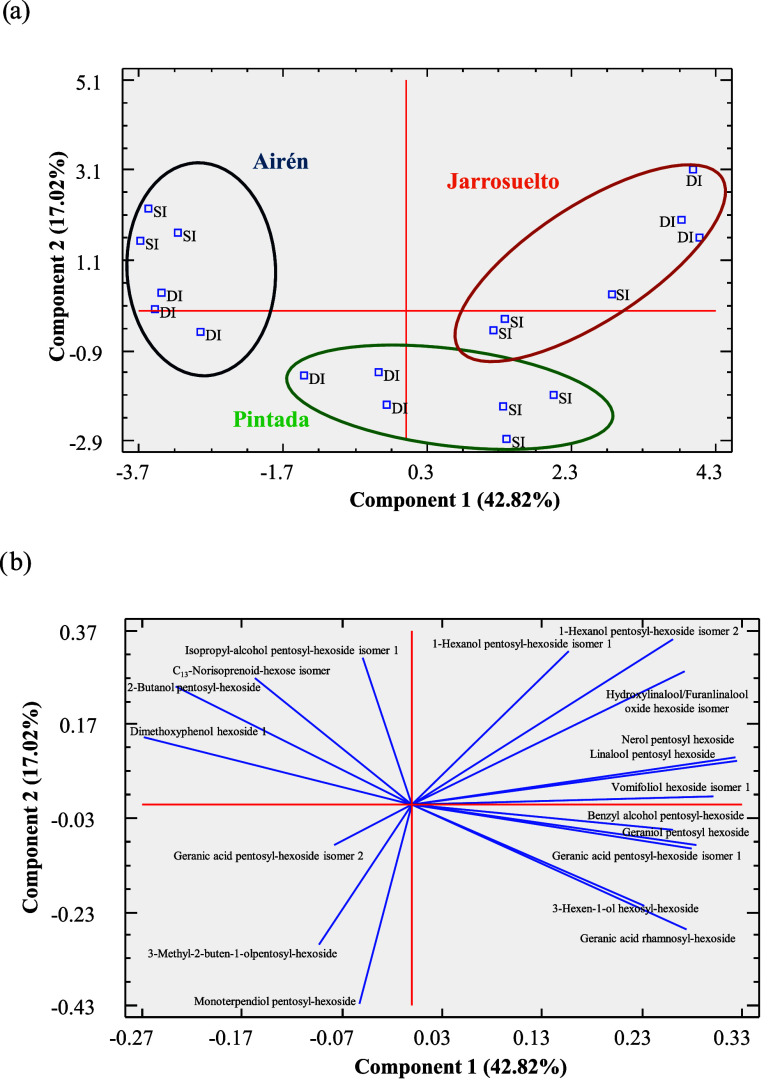
Principal component analysis (PCA) performed using the mean normalized LC/MS signals (three repetitions) of putative glycosidic aroma precursors identified in the white grape varieties. (a) Projection of grape samples in the plane is formed by the two main components: varieties and irrigation regimes; (b) projection of putative glycosidic aroma precursors in the plane is formed by the two main components.

The projection of samples onto the factor-plane delineates three distinct groups based on grape variety, specifically Airén, Pintada, and Jarrosuelto, as illustrated in Figure 1a. PC1 primarily distinguishes Airén from Pintada and Jarrosuelto, while also revealing a differentiation between samples classified as SI and DI within each group. These findings are consistent with the results presented in the two-way ANOVA data in Table 1, which indicate that the glycosidic aroma precursors are predominantly influenced by the variable “variety”. Furthermore, this corroborates previous research on red grape varieties subjected to varying water stress conditions.^32^ As depicted in Figure 1a, there exists a clear separation between the recovered varieties (Pintada and Jarrosuelto) and Airén, highlighting greater divergence from the traditional variety than between the two recovered ones. Additionally, the separation among the different water stress levels within the recovered varieties was more evident, aligning with the smaller number of statistically significant differences observed in the *t* test analysis (Table 2).

The main variables positively contributing to PC1 were linalool pentosyl-hexoside and nerol pentosyl-hexoside, while dimethoxyphenol hexoside and 2-butanol pentosyl-hexoside contribute negatively (Table S3). The variables with the strongest positive loadings on PC2 were the two 1-hexanol pentosyl-hexoside isomers, whereas negative loadings were associated with monoterpendiol pentosyl-hexoside and 3-methyl-2-buten-1-ol pentosyl-hexoside (Table S3). Based on this distribution, Airén samples are positioned in the negative PC1 region, primarily influenced by dimethoxyphenol hexoside and 2-butanol pentosyl-hexoside (Figure 1b). Conversely, Jarrosuelto samples cluster in the positive PC1 area, mainly influenced by nerol pentosyl-hexoside and linalool pentosyl-hexoside, together with vomifoliol hexoside isomer (Figure 1b). Pintada occupies the negative PC2 space, which is predominantly associated with the monoterpendiol pentosyl-hexoside signal. In light of these results, it can be highlighted that, in Airén, benzenoids were the predominant group, mainly driven by dimethoxyphenol hexoside, while Pintada showed a distinct profile characterized by higher monoterpenol glycosides (e.g., linalool-, nerol- and geraniol-derived pentosyl-hexosides) compared with Airén, particularly under survival irrigation. Other terpenoid derivatives (geranic acid glycosides) were also more abundant in Pintada, suggesting a varietal specificity for these compounds and finally Jarrosuelto was characterized by high levels of monoterpenol-related compounds, especially hydroxylinalool/furanlinalool oxide hexoside isomers

A differentiation among the three varieties was observed based on their aroma precursor profiles, driven in part by the presence of two metabolites that appeared to be variety-specific (Table 3). One of these metabolites is the signal corresponding to a putative isopropyl alcohol pentosyl-hexoside isomer, eluting at *R*_t_ = 4.04 min, which was detected exclusively in Jarrosuelto. The other metabolite is a C_13_-norisoprenoid hexose–hexose, eluting at *R*_t_ = 12.56 min, which was found only in Airén. Notably, a recent study reported an isomeric compound with the molecular formula C_25_H_42_O_13_ in Grenache grape extract, identified as a monoterpenol-pentose-pentose–pentose derivative, although the aglycone fragment could not be detected.^76^ The mean signal intensities of these metabolites detected in each variety are shown in Table 3 and their MS/MS spectra and structures are shown in Figure 2. These metabolites may therefore serve as potential varietal markers, useful for detecting the presence of these grapes, for example, in blended musts or wines^31^ although it would be advisible to study, among others, the effect of the harvest season.

**Figure 2:**
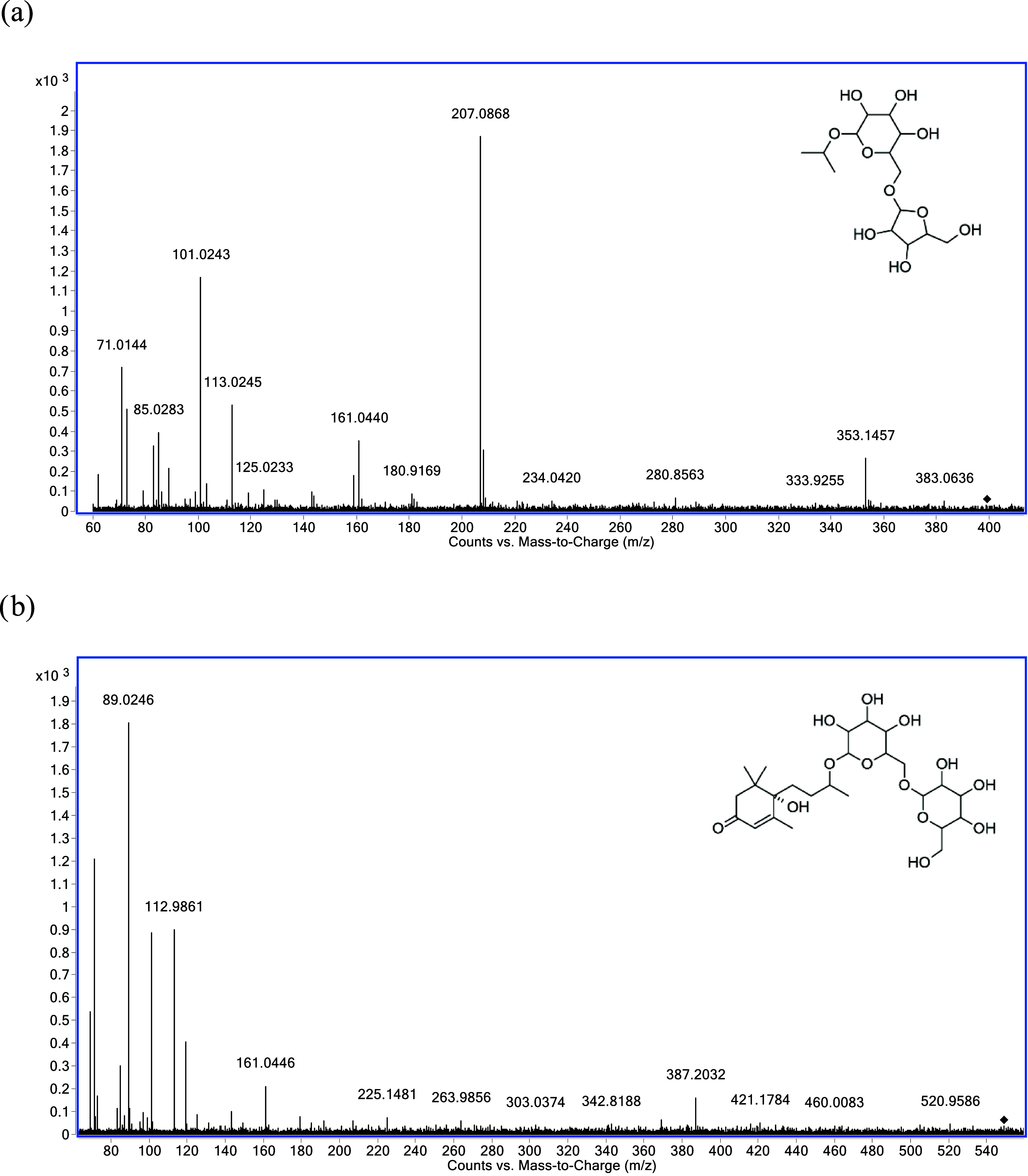
MS/MS spectra of the putative glycosidic aroma precursors identified as variety markers: (a) Isopropyl alcohol pentosyl-hexoside, precursor ion [M + HCOO]^−^ at *m*/*z* 399.1508 (Jarrosuelto); (b) C_13_–Norisoprenoid hexose–hexose, precursor ion [M – H]^−^ at *m*/*z* 549.2553 (Airén).

**Table 3:** Intensity of Signals/g_s+p_ of Variety Markers Identified in Airén and Jarrosuelto^a^

	Airén			Jarrosuelto		
	survival	deficit	*F* ^p‑value^	survival	deficit	*F* ^p‑value^
Markers						
isopropyl alcohol pentosyl-hexoside isomer 2	n.d.	n.d.		15,231 ± 3152a	62,814 ± 7437b	104.12***
C_13_–Norisoprenoid hexose–hexose	30,438 ± 4141a	28,367 ± 3231a	0.47	n.d.	n.d.	

a The mean values (*n* = 3) are shown with their standard deviation. For each compound, different letters indicate significant differences between both water stress regime according to *t* test (***p value <0.001) and typed in bold. n.d.: not detected.

Findings of the present study indicate that the response to water stress varies primarily according to the variety. In comparison to Airén, Pintada, and Jarrosuelto grapes exhibited elevated levels of glycosidic aroma precursors, including benzenoids, aliphatic alcohols, norisoprenoids, monoterpenols, and terpenoids, suggesting a significant enological potential. Furthermore, the Pintada variety demonstrated a greater accumulation of these metabolites in the berries when cultivated under survival stress conditions than the other varieties. Two specifics variety markers have been identified: a norisoprenoid hexose–hexose derivative exclusive to Airén, and an isopropyl alcohol pentosyl-hexoside isomer found only in Jarrosuelto. Thus, within the conditions of this growing season, the differentiation among these varieties could be based on the presence of these intact glycosidic aroma precursors.

The research highlights that a comprehensive understanding of the intact profile of glycosylated aroma precursors is essential for assessing the aromatic potential of grape varieties. Thus, the recovered varieties Pintada and Jarrosuelto could offer a significant opportunity to diversify the current viticulture in Spain, since grapes exhibit a promising enological trait under water stress that would have to be ratified through the study of the proportion of these precursors can be released during winemaking.

## Supplementary Material



## Notes

Putative glycosidic aroma precursors identified by UHPLC/QTOF analysis in Airén, Pintada and Jarrosuelto varieties, berry weight (g) of the white grape varieties studied, and principal component analysis (PCA) performed using the normalized signals of glycosidic aroma precursors identified (PDF)
