# Supplementary material for: Study of Intact Glycosidic Aroma Precursors in Recovered Minority White Grape Varieties under Water Stress Conditions
Source: J Agric Food Chem. 2026 Mar 2;74(9):7864–77. doi: 10.1021/acs.jafc.5c17579 (PMC12983324; doi:10.1021/acs.jafc.5c17579)
Supplement: Supplementary file 1 [file jf5c17579_si_001.pdf]

SUPPORTING INFORMATION

STUDY OF INTACT GLYCOSIDIC AROMA PRECURSORS IN RECOVERED MINORITY WHITE GRAPE VARIETIES UNDER WATER STRESS CONDITIONS

Cristina Cebrián-Tarancón,[a] *Mirko De Rosso,*[b] *Annarita Panighel,*[b] *Riccardo Flamini,*[b] *Gonzalo L. Alonso,*[a] *M. Rosario Salinas,*[a] *A. Sergio Serrano,*[c] *Rosario Sánchez-Gómez*[a*]

[a] Cátedra de Química Agrícola, Universidad de Castilla-La Mancha, E.T.S.I. Agronómica y de Montes y Biotecnología (ETSIAMB), Avda. de España s/n, 02071 Albacete, Spain

[b] Council for Agricultural Research and Economics - Research Center for Viticulture & Enology,

Viale XXVIII Aprile 26, Conegliano (TV), 31015, Italy

[c] Instituto Regional de Investigación y Desarrollo Agroalimentario y Forestal de Castilla-La Mancha (IRIAF), Ctra. Toledo-Albacete s/n, 13700 Tomelloso, Spain

[*] Corresponding author: Tel: +34 967 599210, Fax: +34 967 599238,

e-mail: rosario.sgomez@uclm.es

Table S1. Putative glycosidic aroma precursors identified by UHPLC/QTOF analysis in Airén, Pintada and Jarrosuelto varieties.

Glycosidic aroma precursors	Rt (min)	i.d. Score (%)	Mass error (ppm)	Mass (Tgt)	Formula	main MS/MS ion (*m/z*)	main MS/MS ion Fragment
Benzenoids							
Dimethoxyphenol hexoside*	7.66	99.7	-0.7	316.1182	$C_{14}H_{20}O_8$	153.0556	aglycone
Benzyl alcohol pentosyl-hexoside**	13.26	99.3	0.0	402.1526	$C_{18}H_{26}O_{10}$	269.1034	M-hexose
Monoterpenols							
Hydroxylinalool/Furanlinalooloxide hexoside isomer*	14.48	98.6	-0.3	332.1835	$C_{16}H_{28}O_7$	169.1237	aglycone
Monoterpendiol pentosyl-hexoside**	15.28	97.6	-1.9	464.2258	$C_{21}H_{36}O_{11}$	331.1767	M-hexose
Linalool pentosyl-hexoside**	17.22	96.7	-2.2	448.2308	$C_{21}H_{36}O_{10}$	161.0459	frag. hexose
Nerol pentosyl-hexoside**	17.40	99.6	-1.8	448.2308	$C_{21}H_{36}O_{10}$	315.1808	M-hexose
Geraniol pentosyl-hexoside**	17.60	99.0	1.3	448.2308	$C_{21}H_{36}O_{10}$	315.1808	M-hexose
Aliphatic alcohols							
Isopropyl alcohol pentosyl-hexoside isomer 2$^{\Phi}$ **	4.04	98.5	-1.7	354.1526	$C_{14}H_{26}O_{10}$	207.0868	(M-hexose)-CH_3
Isopropyl alcohol pentosyl-hexoside isomer 1**	5.72	99.0	-1.0	354.1526	$C_{14}H_{26}O_{10}$	221.1035	M-hexose
2-Butanol pentosyl-hexoside**	11.63	99.5	-0.2	368.1682	$C_{15}H_{28}O_{10}$	235.1194	M-hexose
3-Methyl-2-buten-1-ol pentosyl-hexoside**	12.78	99.4	0.5	380.1682	$C_{16}H_{28}O_{10}$	247.1181	M-hexose
3-Hexen-1-ol hexosyl-hexoside isomer*	13.19	99.3	0.7	424.1945	$C_{18}H_{32}O_{11}$	261.1338	M-hexose
1-Hexanol pentosyl-hexoside isomer 1**	14.03	92.7	-3.3	396.1995	$C_{17}H_{32}O_{10}$	161.0500	frag. hexose
1-Hexanol pentosyl-hexoside isomer 2**	15.34	96.6	-3.5	396.1995	$C_{17}H_{32}O_{10}$	263.1510	M-hexose
Norisoprenoids							
C_{13}-Norisoprenoid hexose-hexose$^{\Phi}$ **	12.56	99.8	-1.9	550.2625	$C_{25}H_{42}O_{13}$	225.1481	aglycone
C_{13}-Norisoprenoid hexose isomer**	13.44	97.4	1.0	390.2254	$C_{19}H_{34}O_8$	227.1636	aglycone
Vomifoliol hexoside isomer*	13.43	98.8	-0.4	386.1941	$C_{19}H_{30}O_8$	191.0557	frag. M-hexose
Other terpenoids							
Geranic acid pentosyl-hexoside isomer 1*	17.57	98.1	-1.0	462.2101	$C_{21}H_{34}O_{11}$	167.1068	geraniate
Geranic acid rhamnosyl-hexoside*	17.61	99.1	-0.6	476.2258	$C_{22}H_{36}O_{11}$	167.1075	geraniate
Geranic acid pentosyl-hexoside isomer 2*	17.75	99.5	0.1	462.2101	$C_{21}H_{34}O_{11}$	167.1079	geraniate

** Reported in grape by Cebrián-Tarancón et al. 2025.*
***Identification reported in Panighel et al.[31] and the references cited herein.*
$^{\Phi}$ Proposed as white grape varieties markers.

Table S2. Berry weight (g) of the white grape varieties studied.

Variety	Water stress regime	grape (g)
Airén	SI	53.75 ± 2.80
	DI	47.63 ± 4.39
Pintada	SI	31.33 ± 1.08
	DI	42.37 ± 4.69
Jarrosuelto	SI	47.33 ± 3.84
	DI	54.91 ± 0.75

SI: survival irrigation regime; DI: deficit irrigation regime; grape (g) = pulp + skin weight

Table S3. Principal component analysis (PCA) performed using the normalized signals of glycosidic aroma precursors identified.

	**Component 1 (44.82%)**	**Component 2 (17.02%)**
Dimethoxyphenol hexoside	**-0.2694**	0.1436
Benzyl alcohol pentosyl-hexoside	0.2619	-0.0562
Hydroxylinalool/Furanlinalool oxide hexoside isomer	0.2735	0.2816
Monoterpendiol pentosyl-hexoside	-0.0531	**-0.4243**
Linalool pentosyl-hexoside	**0.3257**	0.0915
Nerol pentosyl-hexoside	**0.3235**	0.1014
Geraniol pentosyl-hexoside	0.2841	-0.0874
Isopropyl alcohol pentosyl-hexoside isomer 1	-0.0491	0.3132
2-Butanol pentosyl-hexoside	**-0.2364**	0.2511
3-Methyl-2-buten-1-ol pentosyl-hexoside	-0.0937	**-0.3009**
3-Hexen-1-ol hexosyl-hexoside isomer	0.2333	-0.2181
1-Hexanol pentosyl-hexoside isomer 1	0.1561	**0.3269**
1-Hexanol pentosyl-hexoside isomer 2	0.2617	**0.3518**
C_{13}-Norisoprenoid hexose isomer	-0.1575	0.2696
Vomifoliol hexoside isomer	0.3018	0.0172
Geranic acid pentosyl-hexoside isomer 1	0.2801	-0.0938
Geranic acid rhamnosyl-hexoside	0.2739	-0.2664
Geranic acid pentosyl-hexoside isomer 2	-0.0776	-0.0889

Variables with the highest weight have been typed in bold.
